# TM4SF5-mediated CD44v8-10 splicing variant promotes survival of type II alveolar epithelial cells during idiopathic pulmonary fibrosis

**DOI:** 10.1038/s41419-019-1878-5

**Published:** 2019-09-09

**Authors:** Ji Eon Kim, Hye-Jin Kim, Jae Woo Jung, Dae-Geun Song, Dasomi Park, Haesong Lee, Hyejin Um, Jinsoo Park, Seo Hee Nam, Moonjae Cho, Jung Weon Lee

**Affiliations:** 1 Department of Pharmacy, Research Institute of Pharmaceutical Sciences, College of Pharmacy, Seoul National University, Seoul, 08826 Republic of Korea; 2 Interdisciplinary Program in Genetic Engineering, Seoul National University, Seoul, 08826 Republic of Korea; 3 Systems Biotechnology Research Center, Korea Institute of Science and Technology (KIST), Gangneung-si, Gangwon-do 25451 Republic of Korea; 4 Institute of Medical Science, Department of Biochemistry, School of Medicine, Jeju National University, Jeju, 63243 Republic of Korea

**Keywords:** Cell growth, Stress signalling, Mechanisms of disease, Respiratory tract diseases

## Abstract

Reactive oxygen species (ROS) regulate cell fate, although signaling molecules that regulate ROS hormesis remain unclear. Here we show that transmembrane 4 L six family member 5 (TM4SF5) in lung epithelial cells induced the alternatively spliced CD44v8-10 variant via an inverse ZEB2/epithelial splicing regulatory proteins (ESRPs) linkage. TM4SF5 formed complexes with the cystine/glutamate antiporter system via TM4SF5- and CD44v8-10-dependent CD98hc plasma-membrane enrichment. Dynamic TM4SF5 binding to CD98hc required CD44v8-10 under ROS-generating inflammatory conditions. TM4SF5 and CD44v8-10 upregulated cystine/glutamate antiporter activity and intracellular glutathione levels, leading to ROS modulation for cell survival. *Tm4sf5*-null mice exhibited attenuated bleomycin-induced pulmonary fibrosis with lower CD44v8-10 and ESRPs levels than wild-type mice. Primary mouse alveolar epithelial cells (AECs) revealed type II AECs (AECII), but not type I, to adapt the TM4SF5-mediated characteristics, suggesting TM4SF5-mediated AECII survival following AECI injury during idiopathic pulmonary fibrosis (IPF). Thus, the TM4SF5-mediated CD44v8-10 splice variant could be targeted against IPF.

## Introduction

Idiopathic pulmonary fibrosis (IPF) is a chronic interstitial lung disease with fibrotic remodeling leading to respiratory failure. IPF is characterized by abnormal cell fates, enriched extracellular matrix (ECM), elevated levels of reactive oxygen species (ROS), and eventual destruction of lung architecture^1^. However, it is still unclear how these characteristics correlate with molecular regulation. Damage to the alveolar epithelium by genetic and environmental factors leads to activation of myofibroblasts expressing α-smooth muscle actin (α-SMA) and subsequent secretion and deposition of ECM components characteristic of IPF development^2^. Molecular links between injury and dysfunction of alveolar epithelium, and activation of myofibroblasts, include the profibrotic cytokine TGFβ1^3,4^ and ROS generated by nicotinamide adenine dinucleotide phosphate-reduced (NADPH) oxidase (NOX)^5^. ROS can also activate TGFβ1 signaling to promote fibrosis^6^. ROS generated from infectious disease, trauma, toxins, drugs, and radiation directly and indirectly contributes to fibrosis via inflammatory responses. Cytokines and growth factors produced by inflammation also contribute to excessive ROS generation^5^. Intracellular levels of ROS can either promote or inhibit cell death in a hormetic manner^7^. Although ROS accumulation in lung epithelial cells is critical for IPF development, the molecular mechanism of ROS modulation in lung epithelia remains unelucidated.

Elevated TGFβ1 signaling and ROS accumulation in an inflammatory environment following epithelial injury leads to aberrant ECM production and deposition^8^. Aberrant ECM synthesis and deposition involved in IPF affects cell fates of alveolar epithelial cells (AECs); type I AECs (AECI) undergo apoptosis, and type II AECs (AECII) are hyperplasic and hypertrophic during IPF^9^. AECII function as facultative stem cells of alveolar epithelium leading to AECI regeneration in response to epithelial injury during IPF^10^. Pathological inflammation along with intracellular ROS accumulation is critically involved in pulmonary fibrosis^11^. Therefore, the identification of a hormetic regulator of intracellular ROS levels in response to pathological inflammation is essential for prevention and treatment of inflammatory diseases such as fibrosis.

Transmembrane 4 L six family member 5 (TM4SF5) is an *N*-glycosylated membrane protein with four transmembrane domains that forms massive protein complexes on the cell surface at TM4SF5-enriched microdomains (T_5_ERMs)^12^ like tetraspanins. TM4SF5 interacts with the EGF receptor and integrin α5^13^ and binds to CD151 and CD63 tetraspanins^14^. TM4SF5 expression is associated with liver fibrosis in CCl_4_-treated animals^15^ and hepatic cancer^16^. Therefore, TM4SF5 has potential roles in the development of pathological conditions in response to chronic inflammation. In addition, further investigation is warranted to determine if TM4SF5 modulates intracellular ROS during pulmonary fibrosis.

This study examined TM4SF5 function in the regulation of intracellular ROS during IPF development. We hypothesized that TM4SF5 could be a signaling-hub molecule in T_5_ERMs^12^, leading to dynamic activation of the xc^−^ system consisting of CD98hc and xCT (SLC7A11), a cystine/glutamate antiporter, in response to ROS-generating stimuli. TM4SF5 could therefore control intracellular ROS levels and AEC fates during IPF. We found that increased TM4SF5 expression in lung epithelial cells, but not hepatocytes, induced a CD44v8-10 alternatively spliced variant and formed complexes with the xc^−^ system via ROS-induced membrane localization of CD98hc, depending on TM4SF5-induced CD44v8-10. This led to hormetic intracellular glutathione (GSH) and ROS modulation. Furthermore, TM4SF5-mediated effects on ROS regulation and fibrotic phenotypes were observed in primary AECII from bleomycin-treated wild-type mice, but not from *Tm4sf5*-knockout mice. Thus, TM4SF5 and CD44v8-10 could be promising therapeutic targets for the prevention and treatment of IPF.

## Results

**Fig. 1:**
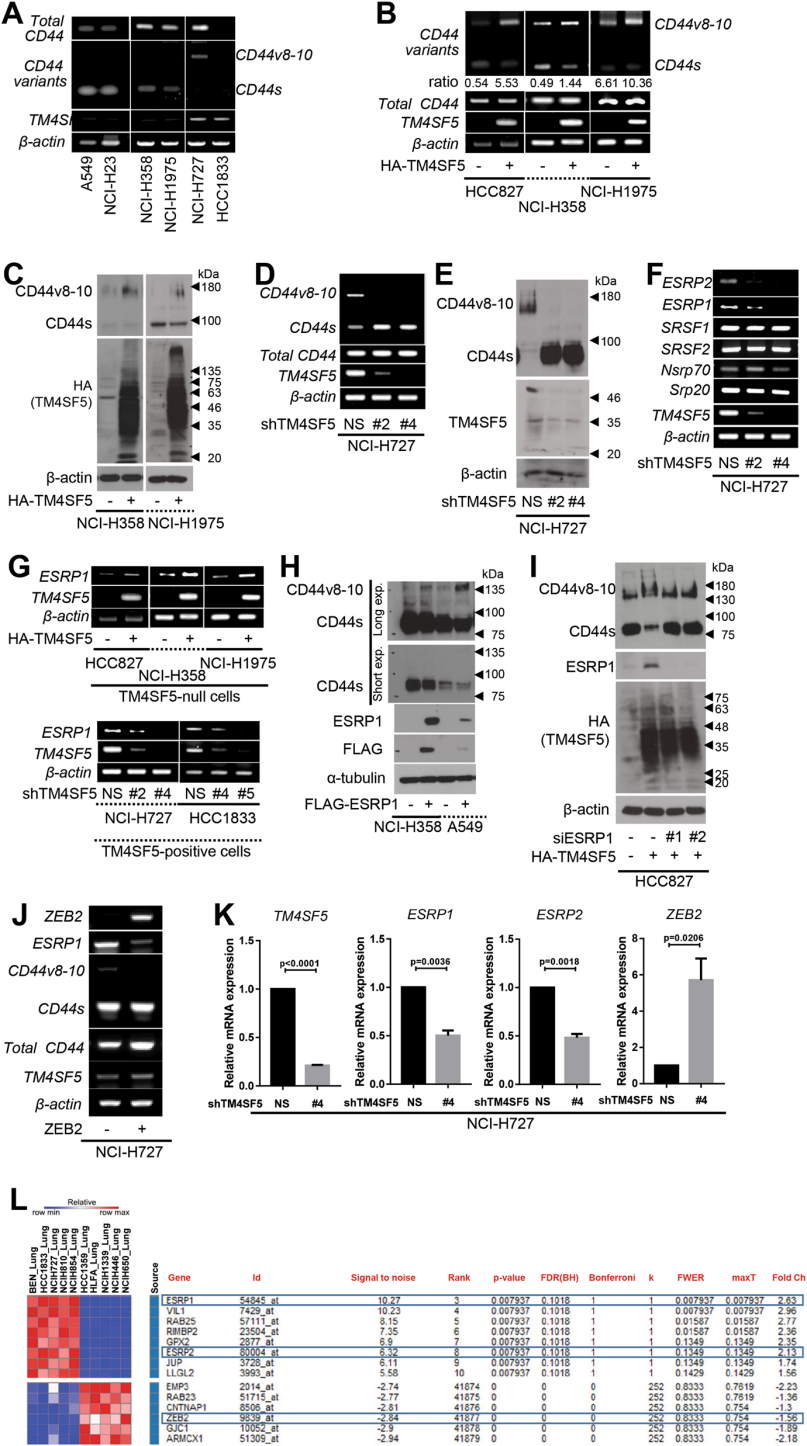
TM4SF5 expression induced alternative splicing variant CD44v8-10 depending on ZEB2 and ESRPs. **a**–**c** Different lung epithelial cells transduced without or with HA-TM4SF5 retrovirus were processed for RT-PCR (**a**, **b**) or western blot analysis (**c**). **d**–**g** Diverse lung epithelial cells transduced with shRNA for a control (NS) or TM4SF5 sequences (shTM4SF5, #2 or #4, Table 1), or transduced with control (−) or HA-TM4SF5 plasmid-containing retrovirus ( + ) were processed for RT-PCR (**d**, **f**, and **g**) or western blot analysis (**e**). **h**–**j** Lung cells transduced with control (−) or FLAG-ESRP1 (**h**), HA-TM4SF5 retrovirus, siRNA (−) or siESRP1 (#1 or #2 sequence, Table 1) (**i**), and control plasmid- (−) or ZEB2 plasmid-containing retrovirus were processed for western blot analysis (**h**, **i**) or RT-PCR (**j**). **k** NCI-H727 cells were transduced with control (NS) or shTM4SF5-containing lentivirus (#4 sequence, Table 1) prior to qRT-PCR analysis. The *p* values were calculated by two-tailed unpaired Student’s *t*-test. *P* values <0.05 were considered statistically significant. **l** Analysis of TM4SF5-positive lung cell lines from the Cancer Cell Line Encyclopedia (CCLE) for expression levels of the indicated (blue boxed) molecules. Data shown represent three independent experiments. See also Figure S1

Proteomic analysis for TM4SF5-binding proteins in hepatic epithelial cells revealed CD44 interaction with TM4SF5^17^. Because there are different alternatively spliced variants of CD44^18^, we examined if TM4SF5 exhibited relationships with specific CD44 variant forms. We first discovered that mRNA levels of *CD44* variants were minimally expressed in hepatic epithelial cells, in particular a CD44 variant form, *CD44v8-10*, with exons 13–15, in addition to a standard form, *CD44s*, that has exons 1–5 and 16–20 (Fig. S1A). In addition, ectopic TM4SF5 expression in SNU761 hepatocytes failed to result in CD44 variant mRNA expression profile changes (Fig. S1B). However, TM4SF5-positive lung epithelial cells positively correlated with *CD44v8-10* mRNA expression (Fig. 1a). Ectopic TM4SF5 expression in lung epithelial cells increased *CD44v8-10* mRNA levels, decreased *CD44s* mRNA levels, and increased CD44v8-10 protein levels (Fig. 1b, c). Using a splicing luciferase reporter system, we found that TM4SF5 suppression inhibited splicing processes of the intron-containing construct, but not the construct lacking introns (Fig. S1C). Although total *CD44* mRNA levels were not affected by TM4SF5 expression (Fig. S1D, left), suppression of TM4SF5 increased *CD44s* mRNA levels and simultaneously reduced *CD44v8-10* mRNA (Fig. 1d) and protein levels (Fig. 1e). These results suggest a TM4SF5-dependent shift in expression from the CD44s to CD44v8-10 form.

We next examined molecules in lung epithelial cells that could be involved in TM4SF5-mediated *CD44v8-10* mRNA elevation. Among diverse alternative splicing mRNA regulators, *ESRP1* and *ESRP2* positively correlated with *TM4SF5* mRNA levels (Fig. 1f). Exogenous expression or suppression of TM4SF5 led to enhanced or reduced *ESRP1* mRNA levels, respectively (Fig. 1g). Concomitantly, TM4SF5 induced *CD44v8-10* mRNA (Fig. S1D, right). Suppression of ESRP1 reduced CD44v8-10, but increased CD44s protein levels (Fig. S1E); the opposite effect was observed with ESRP1 overexpression (Fig. 1h). Upon ESRP1 suppression, TM4SF5-overexpression-induced CD44v8-10 protein levels were reduced and CD44s levels were restored (Fig. 1i). This correlation between *ESRP1* and *CD44v8-10* was further inversely linked to *ZEB2* mRNA levels, although *ZEB2* mRNA levels did not affect *TM4SF5* and total *CD44* mRNA levels. These indicated that ZEB2 is downstream of TM4SF5 but upstream of ESRP1 and CD44v8-10 (Fig. 1j). In addition, suppression of *TM4SF5* in lung epithelial cells increased *ZEB2* and decreased *ESRP1* and *ESRP2* mRNA levels without affecting other splicing regulators (Fig. 1k and S1F). Meanwhile, *ESRP1* mRNA levels were not significant in hepatocytes, but more obvious in lung epithelial cells (Fig. S1G). Correspondingly, certain lung cell lines from the Cancer Cell Line Encyclopedia (CCLE) that highly express TM4SF5 exhibited a positive correlation with ESRP1/2 expression and negative correlation with ZEB2 expression (Fig. 1l). These data thus suggest that TM4SF5/ESRP1-mediated splicing variant CD44v8-10 is mechanistically involved in lung cell homeostasis following TM4SF5-mediated ZEB2 suppression.

**Fig. 2:**
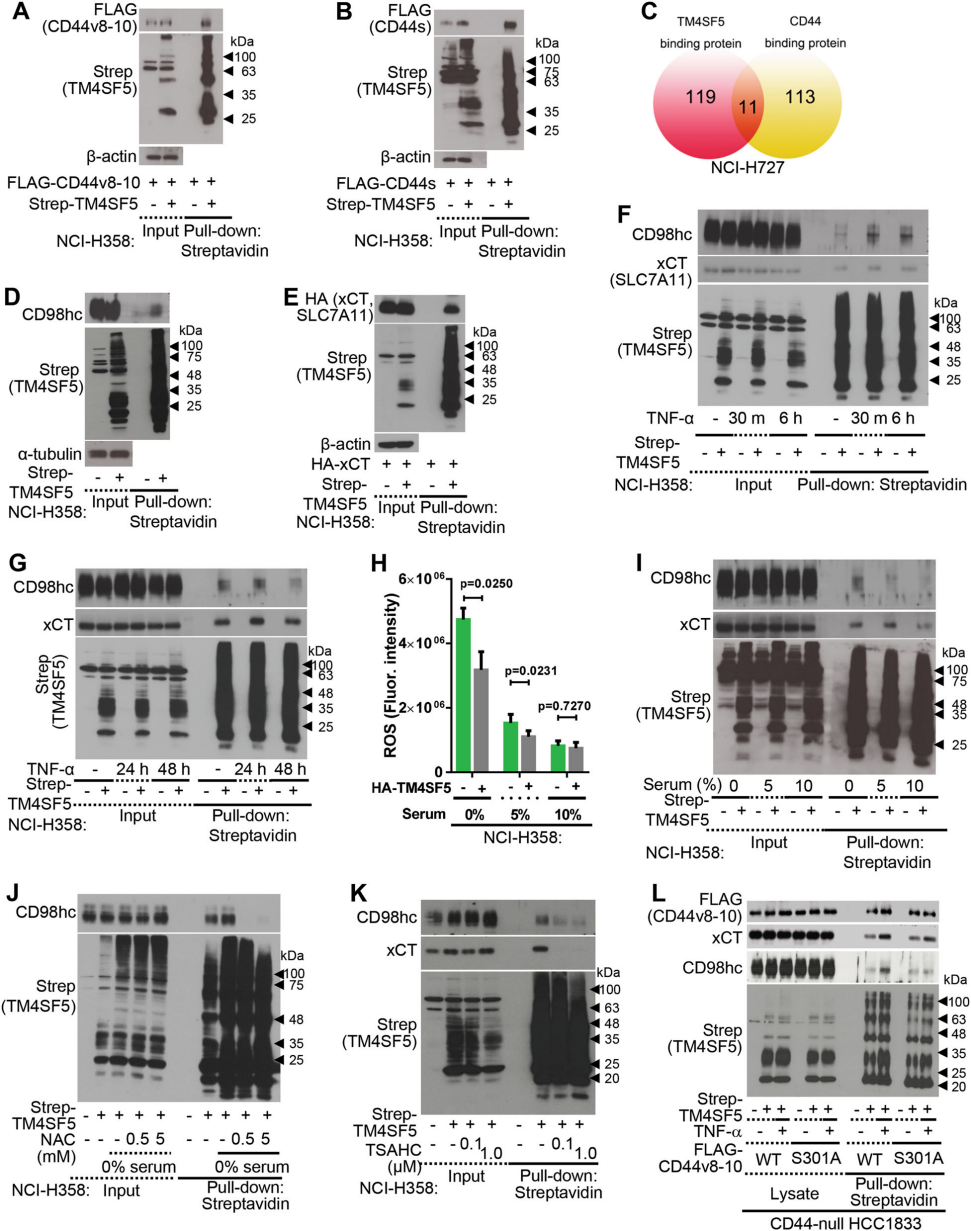
TM4SF5 dynamically induced CD44v8-10 protein complexes with CD98hc and xCT under ROS-generating conditions. **a**, **b** Cells transduced with control Strep (−) or Strep-TM4SF5 plasmid-containing retrovirus were transfected with FLAG-CD44v8-10 plasmid before preparation of whole cell lysate for pull-down with streptavidin-agarose beads and immunoblot analysis. **c** A schematic showing the quantity of molecules binding to TM4SF5 or CD44 during proteomic analysis. **d**, **e** Cells transduced without (**d**) or with (**e**) HA-xCT-encoding retrovirus were transfected with control Strep or Strep-TM4SF5 plasmid. Whole-cell lysates were pulled-down with streptavidin-agarose beads for western blot analysis. **f**, **g** Cells stably transduced with control (−) or Strep-TM4SF5-encoding retrovirus were treated with control vehicle (−) or TNF-α for the indicated times. Whole cell lysates were processed for co-precipitation analysis. **h** Cells transduced with control (−) or HA-TM4SF5 retrovirus were kept in different serum concentrations (0, 5, or 10%) before measurement of DCF-DA fluorescence to stain for intracellular ROS. **i**, **j** Cells transduced with control (−) or HA-TM4SF5 retrovirus were kept in different serum concentrations without (**i**) or with NAC treatment (**j**). Whole-cell lysates were processed for co-precipitation analysis. **k** Cells transfected without (−) or with Strep-TM4SF5 ( + ) were treated with vehicle DMSO (−) or TSAHC for 2 h. Whole-cell lysates were processed for co-precipitation analysis. **l** Cells were transfected with control (−) or Strep-TM4SF5 ( + ) together with wild-type (WT) or S301A mutant FLAG-CD44v8-10 plasmids, prior to TNF-α treatment (2 ng/ml) for 6 h. Whole-cell lysates were processed for co-precipitation analysis. *P* values were calculated by two-tailed, unpaired Student’s *t*-test. *P* values <0.05 were considered statistically significant. Data represent three isolated experiments. See also Figure S2

Next, we examined how TM4SF5-mediated CD44v8-10 expression affected lung cell homeostasis. Because we previously demonstrated that CD44 binds TM4SF5, resulting in stem cell properties and metastasis of TM4SF5-positive liver cancer cells^17^, we hypothesized that CD44s and CD44v8-10 differentially bind to TM4SF5. However, no differential or competitive TM4SF5 binding of the CD44 forms was found (Fig. 2a, b, and S2A). We then investigated TM4SF5- and CD44-binding proteins via proteomic approaches and found 11 common binding proteins, including CD98hc (Fig. 2c and Table S1). TM4SF5 co-precipitated CD98hc (a heavy chain of CD98) and xCT (i.e., SLC7A11; a light chain for CD98) (Fig. 2d, e), which are the two components of the xc^−^ system, a cystine/glutamate antiporter^19^. Among the transmembrane 4 L six family member isoforms, TM4SF5 bound CD98hc more than TM4SF1 or TM4SF4 did (Fig. S2B). TM4SF5 binding to CD98hc was abolished by *N*-glycosylation-deficiency in TM4SF5 (Fig. S2C). The TM4SF5 C-terminal half (amino acids 91–197) bound CD98hc (Fig. S2D), which was abolished by deletion of the fourth transmembrane domain (TM4) and cytosolic C-terminus (ΔTM4/C). Deletion of the TM4SF5 cytosolic C-terminus led to weakened xCT binding compared with wild-type TM4SF5 (Fig. S2E). These data indicate that TM4SF5 associates with CD98hc mostly through TM4SF5 TM4. Furthermore, another light chain paired with CD98hc, SLC7A5, also bound TM4SF5 (Fig. S2F), indicating that TM4SF5 has a binding capacity for the xc^−^ system.

Next, we hypothesized that ROS-generating cellular events regulate binding between TM4SF5 and the xc^−^ cystine/glutamate antiporter system. Therefore, we used tumor necrosis factor-α (TNF-α), a potent, pleiotropic, pro-inflammatory cytokine that leads to ROS generation in response to cellular injury and inflammation^20^. TNF-α treatment led to transient binding of TM4SF5 with CD98hc, but more stable binding with xCT (SLC7A11) (Fig. 2f, g). In addition, serum starvation increased ROS levels, which were significantly reduced by TM4SF5 expression; this effect was augmented by culture with 10% serum-containing medium (Fig. 2h). Serum deprivation of lung epithelial cells led to a more serum deficiency-sensitive (or -responsive) association of TM4SF5 with CD98hc than with xCT (Fig. 2i), which was abolished by treatment with *N*-acetyl-L-cysteine (NAC), a ROS scavenger (Fig. 2j). CD44-null cells showed protein interactions less responsive to serum deprivation or TNF-α treatment (Fig. S2G and S2H). Also, NOX2/NOX4 overexpression, known ROS generators^5^, promoted TM4SF5-CD98hc binding (Fig. S2I). TM4SF5 binding to CD98hc and xCT was abolished in the presence of a specific TM4SF5 inhibitor, TSAHC [4′-(*p*-toluenesulfonylamido)-4-hydroxychalcone]^21^ (Fig. 2k). Furthermore, an Ala mutation of Ser301 (that exists only in CD44v8-10 but not in CD44s) abolished TNF-α-induced TM4SF5 binding to CD98hc, but not significantly to xCT (Fig. 2l), implicating the importance of the CD44v8-10 variant in TM4SF5-CD98hc complex formation. Thus, these data support a dynamic TM4SF5-CD98hc interaction under ROS-generating conditions that critically involves CD44v8-10.

**Fig. 3:**
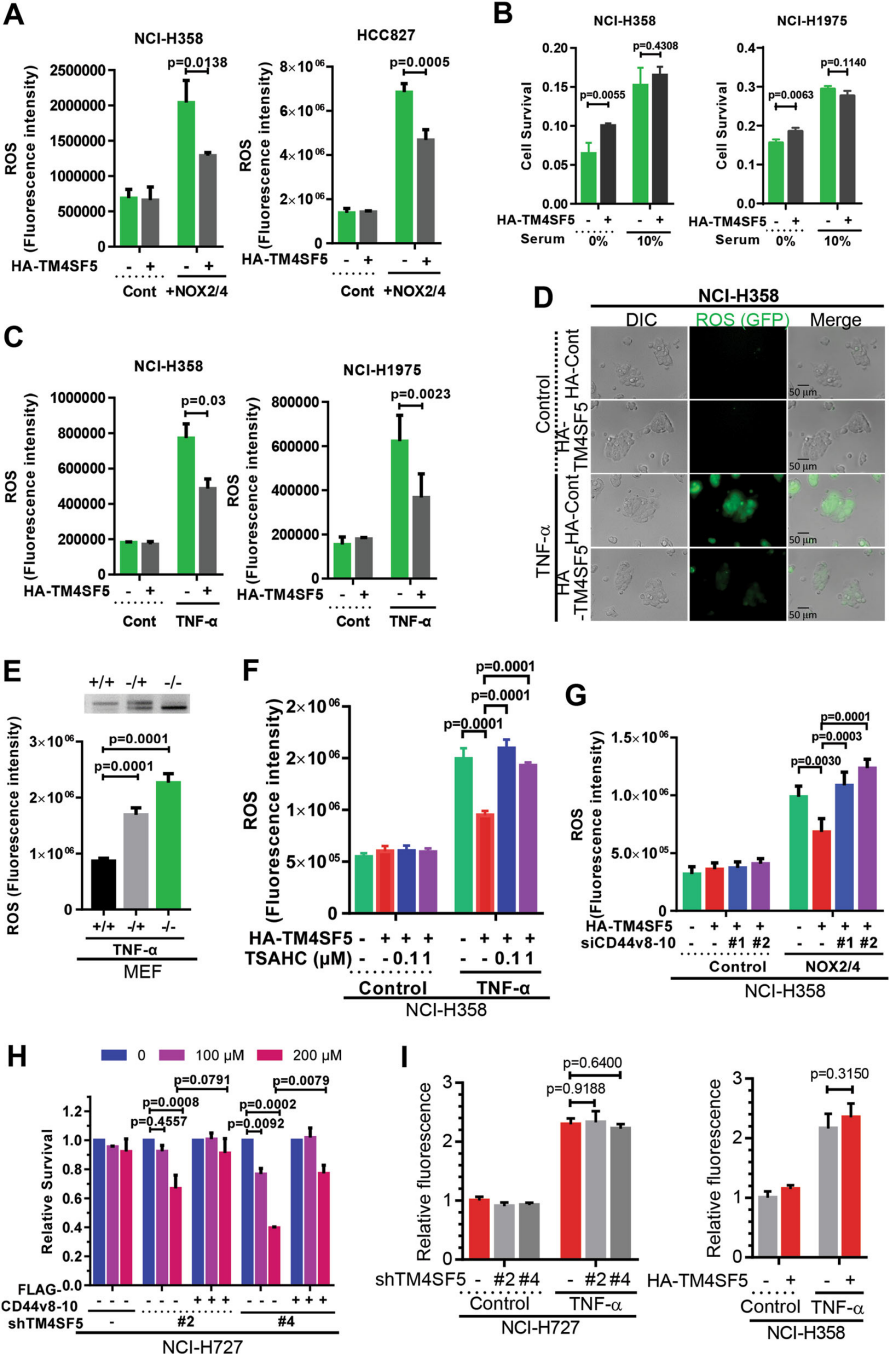
TM4SF5-mediated ROS modulation resulted in increased survival in epithelial cells. **a**, **c** Cells stably transduced with control (−) or HA-TM4SF5 plasmid-containing retrovirus were transfected with NOX2 and NOX4 plasmids (**a**) and subsequently cultured under serum starvation (**b**) or TNF-α treatment (**c**) in parallel to normal control condition (Cont) followed by DCF-DA fluorescence staining for intracellular ROS measurement. *P* values were calculated by two-tailed, unpaired Student’s *t*-test. **d** Cells stably transduced with control (−) or HA-TM4SF5 retrovirus were treated with control vehicle (Control) or TNF-α, followed by fluorescence and phase-contrast imaging. **e** Mouse embryonic fibroblasts (MEFs) from wild-type (+ / +), heterozygous knockout (−/ +), or homozygous knockout (−/−) animals were treated with TNF-α before DCF-DA staining for ROS measurement. **f** Cells were stably transduced without (−) or with HA-TM4SF5 retrovirus with vehicle DMSO (−) or TSAHC treatment, and were concomitantly treated with TNF-α before ROS measurement. **g** Cells stably transduced without (−) or with HA-TM4SF5 retrovirus were transfected with control (−) or NOX2/NOX4 plasmids along with control (−) or siRNA against CD44v8-10 (#1 or #2 sequences, Table 1) before TNF-α treatment and ROS measurement. *P* values were calculated by two-tailed, unpaired Student’s *t*-test. **h** Cells were stably transduced with control (−) or shTM4SF5-encoding (#2 or #4 sequence of TM4SF5, Table 1) lentivirus and then transfected with control (−) or FLAG-CD44v8-10 plasmid before relative cell survival measurement via MTT assay. **i** NCI-H727 cells were treated with shRNAs for a control sequence or to suppress endogenous TM4SF5 (#2 or #4), NCI-H358 cells were transfected with control (−) or HA-TM4SF5 ( + ) plasmids for 48 h, and they were then incubated without (Control) or with TNF-α (2 ng/ml) for 6 h before determination of MitoSOX fluorescence. The *p* values were analyzed by ANOVA with Tukey’s range-test. *P* values <0.05 were considered statistically significant. Data represent three independent experiments. See also Figure S3

We next examined how TM4SF5 modulated intracellular ROS levels in AECs. NOX2/NOX4 overexpression, serum deprivation, and TNF-α treatment increased ROS levels that were partially restored to baseline levels by TM4SF5 overexpression (Fig. 3a–d and Fig. S3A–C). Furthermore, mouse embryonic fibroblasts (MEFs) isolated from wild-type (*Tm4sf5*^*+/+*^), heterozygous (*Tm4sf5*^*−/+*^), and homozygous (*Tm4sf5*^*−/−*^) mice exhibited a gradual increase in ROS levels with *Tm4sf5* gene knockout (Fig. 3e). In addition, TM4SF5 expression decreased ROS levels even with TNF-α treatment, and this effect was reversed by TSAHC pharmacological inhibition of TM4SF5 (Fig. 3f). Furthermore, TM4SF5 overexpression decreased ROS levels even with NOX2/NOX4 overexpression; however, this effect was blocked by CD44v8-10 suppression (Fig. 3g). Thus, intracellular ROS modulation was dependent on TM4SF5 expression. Moreover, TM4SF5 suppression-mediated cell death was partially rescued by CD44v8-10 overexpression (Fig. 3h). Likewise, TM4SF5 overexpression minimized cell death and increased cell survival signaling (i.e., pS^473^Akt) in response to H_2_O_2_ treatment (Fig. S3D and S3E). Meanwhile, TM4SF5 suppression in NCI-H727 cells or overexpression in NCI-H358 cells did not alter the superoxide levels produced by mitochondria following TNF-α treatment (Fig. 3i). Taken together, these data indicate that TM4SF5-mediated expression of the CD44v8-10 variant exerts cellular protection against cytosolic ROS-mediated cytotoxicity.

**Fig. 4:**
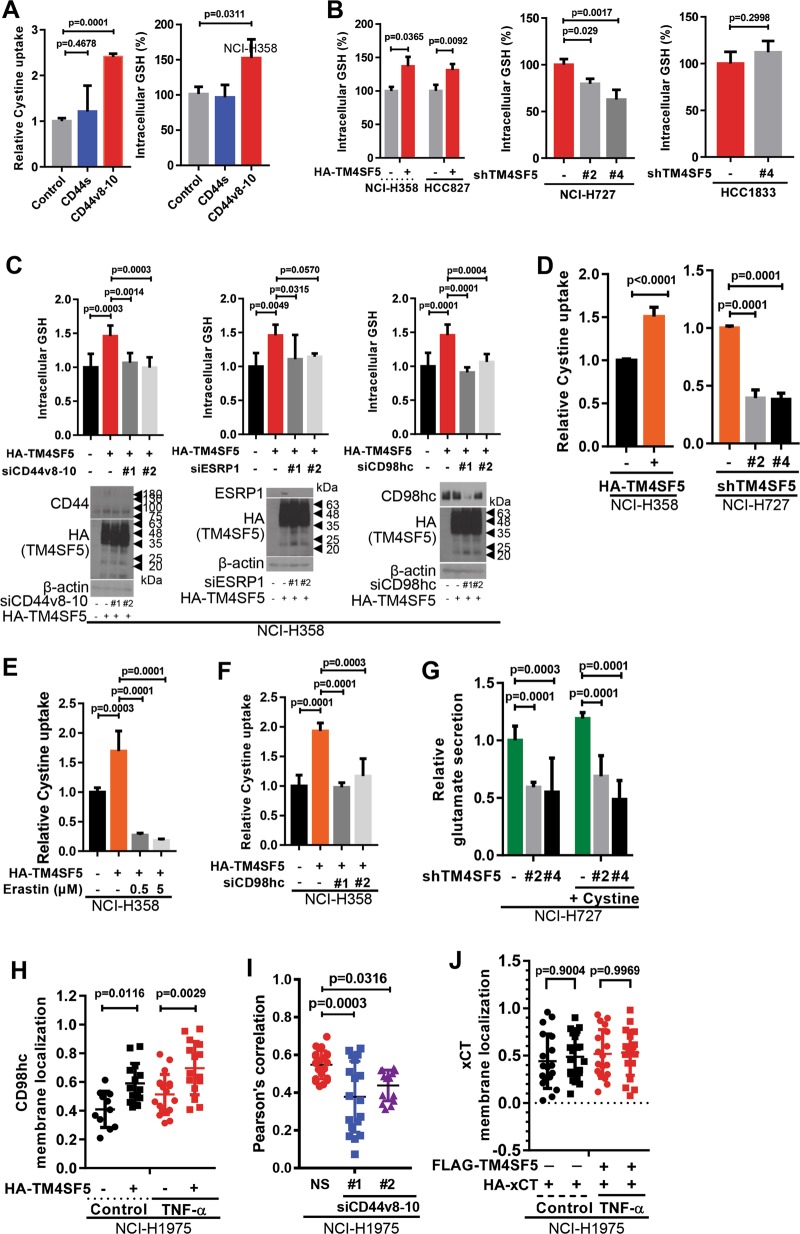
TM4SF5-mediated CD44v8-10 expression increased CD98hc enrichment at the plasma membrane for intracellular GSH-mediated ROS modulation. **a** Cells were transfected with control, CD44s, or CD44v8-10 plasmids, and intracellular GSH levels were measured. **b**, **c** Cells were transduced with the indicated retrovirus and/or lentivirus to target certain sequences of molecules (Table 1) and intracellular GSH levels were measured. Another set of cells was also processed for immunoblot analysis (bottom in **c**). **d**–**f** Cells transduced with different retrovirus or lentivirus without (**d**, **f**) or with erastin treatment (**e**) were processed for cystine uptake analysis. **g** Cells stably transduced with control (−) or shTM4SF5 (#2 or #4 sequence, Table 1) lentivirus were treated without or with cystine in glutamate-free culture media before glutamate secretion measurement. **h**–**j** Cells stably transduced with HA control (−) or HA-TM4SF5 retrovirus (**h**), transfected with siRNA for control (NS) or siCD44v8-10 sequence (#1 or #2, Table 1, **i**), or transfected with FLAG-TM4SF5 and HA-xCT plasmids, were treated without (**i**) or with vehicle (control) or TNF-α (**h**, **j**). Quantitative immunofluorescent images were analyzed for CD98hc (**h**) or xCT (**j**) localization at the plasma membrane or Pearson’s values for co-localization between TM4SF5 and CD98hc at the plasma membrane (**i**). The *p* values were analyzed by ANOVA with Tukey’s range-test. *P* values <0.05 were considered statistically significant. Data represent three isolated experiments. See also Figures S4 and S5

Next, we investigated how TM4SF5-related molecules regulated the xc^−^ systems for intracellular ROS modulation. We first examined if the TM4SF5 modified GSH levels. TM4SF5 substantially increased intracellular GSH levels compared with TM4SF1 and TM4SF4 (Fig. S4A). Interestingly, unlike CD44s, overexpression of CD44v8-10 enhanced cystine uptake and intracellular GSH levels (Fig. 4a). TM4SF5 overexpression or suppression increased or decreased GSH levels, respectively, in CD44-positive lung epithelial cells, whereas CD44-null lung epithelial cells did not exhibit GSH-level changes even after TM4SF5 suppression (Fig. 4a, b). GSH levels were also downregulated by CD44v8-10 suppression (Fig. 4c). Furthermore, TM4SF5 overexpression or suppression increased or decreased cystine uptake, respectively (Fig. 4d), which is responsible for intracellular GSH levels^22^. A specific inhibitor against the xc^−^ system, erastin, blocked TM4SF5-mediated cystine uptake (Fig. 4e), as did CD98hc suppression (Fig. 4f). TM4SF5 suppression in cells under glutamate-free culture media decreased glutamate secretion, independent of additional cystine treatment (Fig. 4g). TM4SF5-dependent secretion of glutamate might occur in response to cellular stress, because glutamate levels in whole extracts prepared from cells in a normal culture condition were comparable independent of TM4SF5 expression (Fig. S4B).

We then investigated if the xc^−^ system could be stabilized by TM4SF5 and CD44v8-10, because TM4SF5 has established roles in membrane trafficking and translocalization with other binding partners^13^. We showed that interaction between TM4SF5 and xCT was independent of ROS-generating stimuli, whereas between TM4SF5 and CD98hc was dynamically modulated by ROS-generating stimuli. Therefore, we analyzed TM4SF5-dependent translocation of CD98hc to the plasma membrane upon ROS generation. We demonstrated that TM4SF5 expression increased translocation of CD98hc to the plasma membrane (–proximal) regions, which was further enhanced by TNF-α-mediated ROS generation (Fig. 4h and Fig. S5A). Suppression of CD44v8-10 reduced co-localization between TM4SF5 and CD98hc on plasma membrane (–proximal) regions (Fig. 4i and Fig. S5B). Meanwhile, membrane localization of xCT (SLC7A11; another component of the xc^−^ system) in the basal or TNF-α-treated conditions did not depend on TM4SF5 expression (Fig. 4j and Fig. S5C). Thus, TM4SF5 and TM4SF5-mediated CD44v8-10 facilitated membrane localization of CD98hc upon ROS generation.

**Fig. 5:**
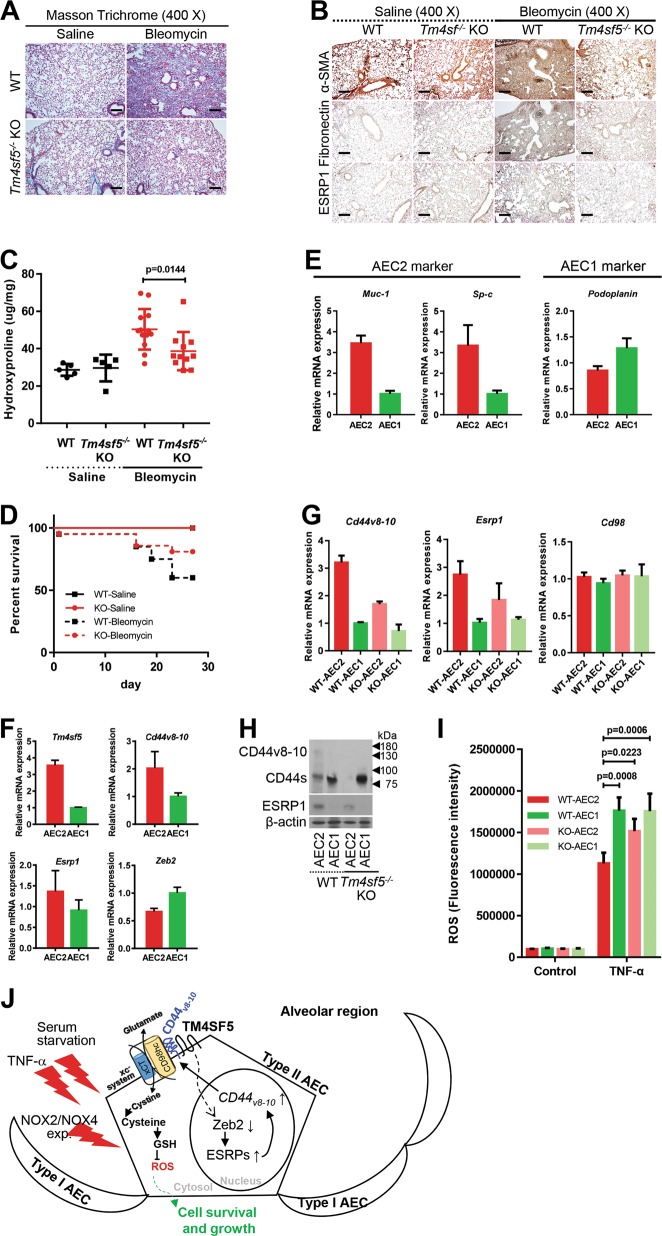
TM4SF5 expression was critical for AECII survival during bleomycin-induced pulmonary fibrosis. **a**, **b** Lung tissues from control saline or bleomycin-treated wild-type (WT) or *Tm4sf5*^−/−^-knockout mice were analyzed for collagen levels by Masson Trichrome staining (**a**), immunohistochemistry for α-SMA and fibronectin (**b**), or hydroxyproline analysis (**c**). **d** The survival rates were plotted for WT or *Tm4sf5*^−/−^ mice treated with either control saline or bleomycin. **e**, **f** AECI or AECII cultures were prepared from WT mice and analyzed for AEC biomarkers (**e**) and *Tm4sf5*-related mRNAs (**f**). **g**, **h** Primary AECI or AECII cells were prepared from lung tissues of WT or *Tm4sf5*^−/−^ mice and processed for qRT-PCR (**g**) or western blot analyses (**h**). **i** The primary cells in (**g**) were treated with control vehicle or TNF-α, followed by DCF-DA staining for ROS. Data represent three isolated experiments. **j** The mechanistic working model. Elevated TM4SF5 expression leads to lower ZEB2 and higher splicing factor ESRP1/2 levels, leading to induction of alternative splicing variant CD44v8-10, which replaces the CD44s form. TM4SF5 and the TM4SF5-induced CD44v8-10 variant form a protein complex along with CD98hc and xCT for cystine uptake and glutamate secretion. This leads increased intracellular GSH levels for hormetic regulation of intracellular ROS levels and eventual survival or protection of AECII from cytotoxic ROS stimuli, such as serum starvation, TNF-α treatment, or NOX2/NOX4 expression

Next, we explored the physiological function of TM4SF5-mediated regulation of intracellular ROS in lung epithelial cells of mice treated with bleomycin to induce pulmonary fibrosis. We postulated that TM4SF5 and its associated molecules were critically involved in pulmonary fibrosis. Intratracheal injection of bleomycin was administrated to wild-type and *Tm4sf5*-knockout (*Tm4sf5*^−/−^) mice to induce pulmonary fibrosis. Bleomycin treatment disrupted alveolar epithelial architecture with collagen enrichment in wild-type mice, whereas *Tm4sf5*^*−/−*^ mice displayed a decreased phenotype (Fig. 5a). Immunostainings for the fibrotic biomarkers α-SMA, fibronectin, and ESRP1 revealed fibrotic lungs in wild-type mice; this observation was minimized in *Tm4sf5*^−/−^ mice (Fig. 5b). Bleomycin treatment also increased hydroxyproline levels that are required for collagen crosslinks in wild-type mice, but this characteristic was not prevalent in *Tm4sf5*^−/−^ mice (Fig. 5c). *Tm4sf5*^−/−^ mice also exhibited increased survival rates after bleomycin treatment compared with wild-type (Fig. 5d). Primary AEC cultures were analyzed for biomarkers of AECI or AECII to evaluate further the AEC type involved in the TM4SF5-protective effects in response to bleomycin-induced pulmonary fibrosis (Fig. 5e). A positive correlation was observed among *Tm4sf5*, *Cd44v8-10*, and *Esrp1* mRNA expression levels and an inverse correlation was detected with *Zeb2* mRNA levels in AECII, but not AECI (Fig. 5f). Furthermore, primary AECII from *Tm4sf5*^−/−^ mice showed lower *Cd44v8-10* and *Esrp1* mRNA levels compared with wild-type mice, whereas AECI did not show differential corresponding mRNA levels between wild-type and *Tm4sf5*^−/−^ mice (Fig. 5g). Likewise, CD44v8-10 and ESRP1 protein levels were higher in AECII from wild-type mice compared with *Tm4sf5*^−/−^ mice, but the standard form, CD44s, was mostly expressed in AECI without ESRP1 expression from both wild-type and *Tm4sf5*^−/−^ mice (Fig. 5h). TNF-α treatment of primary AEC resulted in decreased ROS levels in wild-type AECII, but not AECI or AEC from *Tm4sf5*^−/−^ mice (Fig. 5i), suggesting no ROS-regulatory roles of TM4SF5 in AECI. Therefore, TM4SF5 mediates hormetic levels of intracellular ROS as demonstrated by the bleomycin-induced pulmonary fibrosis model.

## Discussion

This study revealed that TM4SF5 expression in lung epithelial cells induces mRNA splicing for the CD44v8-10 variant via decreased ZEB2 and, in turn, increased ESRPs. This promoted plasma membrane translocation and stabilization of CD98hc and subsequent protein complex formation with xCT to support cystine uptake and glutamate secretion for intracellular GSH-mediated ROS modulation, leading to AEC survival. However, this TM4SF5-mediated mechanism and its corresponding phenotype were observed in primary AECII, but not in AECI, in a bleomycin-induced pulmonary fibrosis animal model. Therefore, this study suggests that TM4SF5-dependent protective effects are important for IPF development (Fig. 5j), and further proposes TM4SF5 and CD44v8-10 as promising therapeutic targets against IPF.

This study revealed that CD44v8-10 variant levels depended on TM4SF5-mediated lower ZEB2 expression and, in turn, TM4SF5-dependent ESRP1/2 expression. Among diverse splicing factors, ESRP1/2 was responsible for TM4SF5-induced CD44v8-10 mRNA splicing as a replacement for the CD44s form. It has also been observed that ESRP-regulated exons overlap exons that exhibit cell type-specific differences largely in epithelial cells compared with mesenchymal cells that alternate during the epithelial–mesenchymal transition (EMT)^23^. Indeed, TM4SF5 is involved in EMT, leading to a loss of contact inhibition in liver cancer cells^16^ and resistance against anti-cancer drugs including paclitaxel or gefitinib^24,25^. Therefore, as a membrane protein, TM4SF5 also triggers EMT-related stem cell properties that initiate tumor formation at low cell numbers as well as circulating tumor cell properties^17^. Furthermore, the TM4SF5-mediated CD44v8-10 induction occurred in primary AECII, but not AECI. Given that AECII can function as facultative stem cells in alveolar epithelium, leading to regeneration of AECI following epithelial injury^10^, TM4SF5 may promote AECII survival and proliferation under ROS-generating, pro-inflammatory conditions to compensate for AECI loss during IPF.

The hormetic regulation of ROS is critical for cellular homeostasis^26^. We discovered that TM4SF5-mediated induction of the alternative splicing variant, CD44v8-10, led to intracellular ROS modulation via formation of TM4SF5/CD44v8-10/xc^−^ system protein complexes. The xc^−^ system consisting of CD98hc and xCT (SLC7A11) influxes cystine and effluxes glutamate, which in turn regulates intracellular GSH and ROS levels in gastric cancer cells^27^. Here, we further elucidate the ROS-mediated regulatory circuit by the xc^−^ system, depending on TM4SF5, as follows: (1) TM4SF5 specifically caused alternative splicing variant CD44v8-10 replacing CD44s via reduced ZEB2 and increased ESRP1/2 expression; (2) TM4SF5 promoted dynamic plasma membrane translocation of CD98hc in response to ROS-generating stimuli; (3) TM4SF5-CD98hc binding dynamically depended on the presence of CD44v8-10, whereas TM4SF5 constitutively bound CD44s and xCT; (4) TM4SF5 and CD44v8-10 expression induced xc^−^ system activation for intracellular GSH and ROS level modulation; and (5) TM4SF5-mediated effects were observed in AECII, not AECI, during IPF. Stabilization of xCT by CD44v8-10 was exhibited in a gastric cancer model^27^, contradictory to this study showing that CD44v8-10 was required for TM4SF5-dependent plasma membrane translocation of CD98hc. This difference could be due to different cell types, pathological states, and/or TM4SF5 expression levels. Furthermore, this study emphasizes the significance of TM4SF5-induced CD44v8-10 in the following aspects: (1) CD44v8-10 splice variant, but not CD44s, upregulated the xc^−^ system activity for cystine uptake and glutamate secretion; (2) TM4SF5-dependent plasma-membrane enrichment of CD98hc required the CD44v8-10 variant; and (3) dynamic association between TM4SF5 and CD98hc upon ROS-generating stimuli required the Ser301 residue in CD44v8-10 that does not exist in CD44s. Thus, these findings may explain why TM4SF5 expression induces alternative splicing of the CD44v8-10 variant to replace the standard CD44s form in AECII.

TM4SF5 has four transmembrane domains, similar to the tetraspanins^12^. Therefore, TM4SF5 can regulate binding stability, intracellular trafficking, and signaling activity by forming massive protein-protein complexes on the cell surface at T_5_ERMs^28^. TM4SF5 expression results in acquired cancer stem cell properties via a physical association with CD44^17^. TM4SF5 expression is promoted by, and subsequently interacts with, CD133, a cancer cell marker^29^. In addition to CD44 and CD133, TM4SF5 interacts with the EGF receptor and integrin α5 for directional migration^13^. Furthermore, pro-metastatic CD151 tetraspanin binds to TM4SF5 to display a synergistic pro-metastatic role on the cell surface; however, TM4SF5 binds anti-metastatic CD63 and sequesters it from the membrane surface into lysosomes to negate its tumor-suppressive roles^14^. Pathological, pro-inflammatory conditions can thus selectively promote TM4SF5 binding with specific proteins at T_5_ERMs in epithelial cells. The hormetic ROS regulation appeared to be via the TM4SF5-mediated influence on the xCT system in the plasma membrane rather than ROS (especially superoxide) formation by mitochondria because TM4SF5 expression did not cause any changes in mitochondrial ROS levels. ROS-mediated cytotoxicity is involved in IPF development^11^, and survival and hyperplasia of AECII compensates for the loss of injured AECI that is a hallmark of IPF^1,9^. Because TM4SF5-related molecules, including ZEB2, ESRPs, and CD44v8-10, were visibly expressed in AECII, TM4SF5, and CD44v8-10 within T_5_ERMs of AECII can play hyperplasic roles during IPF development. Therefore, TM4SF5-mediated hyperplasia of AECII can be targeted by an anti-TM4SF5 reagent, such as TSAHC^21^, leading to clinical benefits for IPF patients.

## Materials and methods

### Cell culture

A549, NCI-H23, NCI-H358, NCI-H1975, NCI-H727, and HCC1833 human lung epithelial cells and liver epithelial cells were purchased from the KCLB (Seoul National University, Seoul, South Korea). Cells were cultured in RPMI medium supplemented with 10% fetal bovine serum (FBS) (GenDepot, Barker, TX, USA), along with routine monitoring for mycoplasma contamination. For experiments requiring starvation conditions, cells were incubated overnight in culture media lacking FBS. Primary AECs were seeded into collagen-coated plates and cultured in DMEM supplemented with 10% FBS, 1 × GlutaMAX (Gibco, Waltham, MA, USA), 10 mM HEPES (Gibco), and 0.25% bovine serum albumin (GenDepot).

### Viral transduction and plasmid DNA and siRNA transfection

Human TM4SF5 (NM-003963) insert was cloned into retroviral expression vector pBabe-HAII (Addgene, Cambridge, MA, USA), resulting in HA-TM4SF5. NCI-H358, H1975, and HCC827 cells were stably transduced with retrovirus expressing pBabe-HA-TM4SF5. To achieve *TM4SF5* knockdown, shRNAs targeting the human *TM4SF5* sequence (#2 and #4, Table 1) were cloned into lentiviral vector pLKO.1 (Addgene). NCI-H727 and HCC1833 cells were stably transduced with pLKO.1-shTM4SF5-expressing lentivirus. CD44s-pBabe-puro and shCD44-pRRL constructs were purchased from Addgene. Human CD44v8-10 (NM-000610.3) and xCT (NM-014331.4) sequences were cloned from cell line NCI-H727. Plasmid cDNAs were transiently transfected into cell lines using Lipofectamine 3000 reagent (Thermo Fisher Scientific, Waltham, MA, USA) according to the manufacturer’s protocol. Oligonucleotides for siRNA targeting CD44v8-10, ESRP1, or CD98hc were synthesized (Bioneer, Daejeon, South Korea), as shown in Table 1, and transfected using Lipofectamine RNAiMAX transfection reagent (Thermo Fisher Scientific) according to the manufacturer’s protocols.

**Table 1:** shRNA target sequence, siRNA sequence, or RT-PCR primer sequence used in this study

*shRNA target sequence*	*Sequence* (*5*′–*3*′)		
shTM4SF5 #2	ACCATGTGTACGGGAAAATGTGC		
shTM4SF5 #4	CCATCTCAGCTTGCAAGTC		
*siRNA sequence*	*Sequence* (*5*′**–***3*′)		
CD44v8-10 #1	CUACUUUACUGGAAGGUUA(dTdT)		
CD44v8-10 #2	GGAAGAAGAUAAAGACCAU(dTdT)		
ESRP1 #1	CCUUCGAGGUCUUCCCUAU(dTdT)		
ESRP2 #2	GCAGCAAGAUGGAACUUAU(dTdT)		
CD98hc #1	AAUCCUGAGCCUACUCGAAUC(dTdT)		
CD98hc #2	GUUCAAGAGACUUCUCGGGCU(dTdT)		
*Gene*	*Primer sequence* (*5*′–*3*′)		Species
TM4SF5	FWD	ACACCAACCATCTCAGCTTG	Human
TM4SF5	REV	CATCTGGGTCCATTTCGGAG	Human
CD44v8	FWD	TGGACTCCAGTCATAGTATAACGC	Human
CD44v10	REV	CGATTGACATTAGAGTTGGAATCTCC	Human
CD44 (total)	FWD	CGGACACCATGGACAAGTTT	Human
CD44 (total)	REV	GAAAGCCTTGCAGAGGTCAG	Human
CD44 (variant)	FWD	TCCCAGCAGACGAAGACAGTCCCTGGAT	Human
CD44 (variant)	REV	CACTGGGGTGGAATGTGTCTTGGTC	Human
ESRP1	FWD	CTCTCGATATGGAGCCTCTCA	Human
ESRP1	REV	CTGCACCTCCCTTGGCAATA	Human
Srp20	FWD	ATGGAAGAACACTATGTGGCTG	Human
Srp20	REV	GGGACGGCTTGTGATTTCTCT	Human
GAPDH	FWD	CCAGCCGAGCCACATCGCTC	Human
GAPDH	REV	ATGAGCCCCAGCCTTCTCCAT	Human
β-actin	FWD	AGAGCTACGAGCTGCCTGAC	Human
β-actin	REV	AGCACTGTGTTGGCGTACAG	Human
ZEB2	FWD	GCGATGGTCATGCAGTCA	Human
ZEB2	REV	CAGGTGGCAGGTCATTTTCTT	Human
ZEB1	FWD	TTACACCTTTGCATACAGAACCC	Human
ZEB1	REV	TTTACGATTACACCCAGACTGC	Human
SRSF1(ASF/SF2)	FWD	GGAAGACGCGGTGTATGGTC	Human
SRSF1(ASF/SF2)	REV	CACCTGCTTCACGCATGTG	Human
SRSF2(SC35)	FWD	CCCGATGTGGAGGGTATGAC	Human
SRSF2(SC35)	REV	GAGACTTCGAGCGGCTGTAG	Human
NSrp70(NSRP1)	FWD	GAACGTCGAGAGGACATGAGA	Human
NSrp70(NSRP1)	REV	TCACGGTCAGTGTAATGGTTCT	Human
CD44 (total)	FWD	CCGTTGGCTGCTTAGTCACAG	Mouse
CD44 (total)	REV	GATGTGGATGTGCCAGGCT	Mouse
CD44 (variant)	FWD	CGATGGACCGGTTACCATAA	Mouse
CD44 (variant)	REV	TGTCCTGGTTCGCACTTG	Mouse
ESRP1	FWD	CTGTGTCCCGATACGGAGC	Mouse
ESRP1	REV	CTTGATCTGAAGATTGCCAGGG	Mouse
ZEB1	FWD	CGCCATGAGAAGAACGAGGAC	Mouse
ZEB1	REV	CTGTGAATCCGTAAGTGCTCTTT	Mouse
ZEB2	FWD	AAACGTGGTGAACTATGACAACG	Mouse
ZEB2	REV	CTTGCAGAATCTCGCCACTG	Mouse
GAPDH	FWD	GTGGCAAAGTGGAGATTGTTG	Mouse
GAPDH	REV	CGTTGAATTTGCCGTGAGTG	Mouse

### Western blot analysis

Cells were harvested for whole cell lysates using RIPA lysis buffer (50 mM Tris-HCl, pH 7.4, 150 mM NaCl, 0.5% Sodium deoxycholate, 0.1% SDS, and 1% NP-40) or Brij58 lysis buffer (20 mM HEPES, pH 7.4, 150 mM NaCl, 2 mM MgCl_2_, 2 mM CaCl_2_, and 1% Brij58) with protease inhibitor cocktails (GenDepot). The lysates were normalized and immunoblot analysis was performed using the following primary antibodies: CD44 (IM7; 1:1000 dilution), CD98hc (H-300; 1:1000 dilution), β-actin (C4; 1:1000 dilution), AKT (H-136; 1:1000 dilution), pS^473^AKT (C11; 1:1000 dilution) (Santa Cruz Biotechnology, Santa Cruz, CA, USA), ESRP1 (27H12; 1:1000 dilution; Abcam, Cambridge, UK), HA (16B12; 1:1000 dilution; Covance, Princeton, NJ, USA), StrepMAB-Classic conjugated to HRP (1:1000 dilution; IBA), FLAG (M2; 1:1000 dilution), xCT (D2M7A; 1:1000 dilution), ERKs (1:2000 dilution), and pT^202^pY^204^ERKs (1:5000 dilution) (Cell Signaling Technology, Danvers, MA, USA).

### Immunoprecipitation assay

Cells were harvested using Brij58-containing lysis buffer. Whole-cell lysates were precipitated with Pierce Streptavidin-Agarose (Thermo Fisher Scientific) for 4 h at 4 °C. Precipitates were washed three times with ice-cold wash buffer (20 mM HEPES, pH 7.4, 500 mM NaCl, 2 mM MgCl_2_, 2 mM CaCl_2_, and 1% Brij58), one time with ice-cold PBS, and then eluted in 2 × SDS-PAGE sample buffer before immunoblot analysis.

### Immunofluorescence

Cells were seeded onto glass coverslips pre-coated with fibronectin (10 μg/ml; BD Bioscience, San Jose, CA, USA) and allowed to attach overnight. The next day, cells were fixed with ice-cold methanol and stained with the following primary antibodies: FLAG (M2; 1:300 dilution; Cell Signaling Technology), HA (16B12; 1:300 dilution; Covance), and CD98hc (H-300; 1:200 dilution; Santa Cruz Biotechnology). Immunofluorescent images were acquired at room temperature using the C2+ confocal microscope (Nikon) with a normal PMT (Nikon) and a CFI Apochromat Lambda S 60 × NA1.49 oil immersion objective (Nikon) after excitation with 405, 488, and 561 nm laser lines. Confocal images were analyzed using NIS software (Nikon) to obtain Pearson’s correlation.

### Reverse transcription PCR (RT-PCR)

Total RNA was extracted from cells using Qiazol reagent (Qiagen, Hilden, Germany), according to the manufacture’s protocol. Total RNA (500 ng) was reverse transcribed into cDNA using ReverTra Ace qPCR RT master mix (Toyobo, Osaka, Japan). The primers used for PCR are listed in Table 1. Quantitative PCR (qPCR) was performed using specific primers and EvaGreen Q master mix (Cosmogenetech, Seoul, South Korea) and the CFX96 real-time system (Bio-Rad, Hercules, CA, USA). Each sample was run as triplicates. Relative fold abundance of target genes was calculated using the comparative Cr method.

### ROS induction and detection

Cells were treated with TNF-α (2 ng/ml, Peprotech, Princeton, NJ, USA) for 30 min, 6 h, 24 h, or 48 h before co-precipitation processes. For intracellular ROS visualization and determination, cells were incubated with 20 μM DCF-DA (Sigma-Aldrich; St. Louis, MO, USA) in RPMI for 30 min at 37 °C and washed with PBS twice. Fluorescence was visualized using fluorescent microscope (BX51TR, Olympus, Tokyo, Japan) or determined using a microplate reader (SpectraMax; Molecular Devices, San Jose, CA, USA) with an excitation wavelength at 490 nm and an emission wavelength at 520 nm. Intramitochondrial superoxide was measured using MitoSOX Red mitochondrial superoxide indicator (Invitrogen, Carlsbad, CA, USA) following the manufacture’s protocol. Cells treated with control vehicle or TNF-α (2 ng/ml for 6 h) were incubated with 2 μM MitoSOX for 10 min at 37 °C and washed with PBS twice. Fluorescence was measured using the SpectraMax microplate reader with an excitation wavelength at 510 nm and an emission wavelength at 580 nm.

### Cystine uptake assay

NCI-H358 or H727 cells (200,000 cells/well) were seeded into 12-well culture plates. After 24 h, cells were washed twice with pre-warmed PBS, and then incubated for 1 h at 37 °C in amino acid-depleted RPMI media. The medium was replaced with medium containing 40 mCi/mmol L-[3,3′-^14^C]-Cystine (American Radiolabeled Chemicals, St. Louis, MO, USA) and incubated for 10 min at 37 °C. Cells were then washed three times with ice-cold PBS and lysed in 0.1 M NaOH. Cell lysates were added to scintillation fluid, and radioactive counts per min were obtained using a scintillation counter (Tri-Carb 2910 TR; Perkin Elmer, San Jose, CA, USA). All experiments were repeated as three independent biological replicates for each condition.

### Glutamate and glutathione level assays

The Amplex Red glutamate assay kit (Thermo Fisher Scientific) or GSH-Glo glutathione assay kit (Promega, Madison, WI, USA) were used to measure glutamate levels in medium (i.e., glutamate secretion) or for intracellular GSH levels from lysates, respectively. Assays were performed according to the manufacturer’s protocols.

### Cell viability assay

Cells were seeded into 96-well plates (5 × 10^3^ cells/well) and treated with H_2_O_2_ (100 or 200 μM) for 24 h. Cell viability was assessed using an MTT assay. Cells were treated with 0.1 mg/ml MTT solution (Sigma-Aldrich) for 4 h at 37 °C. Crystals were solubilized using DMSO and absorbance was measured at a wavelength of 570 nm in a microplate reader (SpectraMax).

### Bleomycin-induced animal pulmonary fibrosis model

Wild-type C57BL/6 mice (4 weeks old) were purchased from Orient Bio Inc. (Seongnam, South Korea). Global TM4SF5-knockout C57BL/6 mice (*Tm4sf5*^−/−^) were generated by Macrogen (Seoul, South Korea). Briefly, base pairs of genomic TM4SF5 surrounding exon 1 were deleted using the RGEN/Cas9 genetic scissor. The genotypes of both wild-type and *Tm4sf5*^−/−^ mice were determined by PCR analysis of genomic DNA obtained from tail clippings from 3-week-old animals. Male wild-type and *Tm4sf5*^−/−^ mice at 8 weeks old were treated with either saline vehicle for the control or bleomycin (1 mg/kg, Santa Cruz Biotechnology) by intratracheal injection on day 0. Mice were sacrificed on day 28 for histological analysis and hydroxyproline assay.

### Hydroxyproline assay

Total lung collagens were determined using a hydroxyproline assay kit (Sigma-Aldrich). Briefly, 10 mg lung tissue was homogenized and hydrolyzed with hydrochloric acid (HCl, ~12 M) at 120 °C for 3 h. The reaction was mixed and centrifuged at 10,000 × *g* for 3 min. The supernatant was transferred to a 96-well plate and 100 μl chloramine-T/oxidation buffer mixture was added to each for reaction initiation. Diluted DMAB [4-*p*-(dimethylamino) benzaldehyde, Sigma-Aldrich] reagent was added to each sample before incubation for 90 min at 60 °C. Absorbance at 560 nm was measured using a microplate reader (Zenyth 3100; Anthos Labtec Instruments; Wals/Salzburg, Austria).

### Primary AECs isolation and culture

Mice at 6 weeks of age were sacrificed for primary pneumocyte isolation. A 26G cannula with a 10 ml syringe filled with cold PBS was used to puncture through the right ventricle of the heart into the lung for perfusion and complete blood removal. A 22G indwelling cannula with a 2 ml syringe was inserted into the trachea and 2 ml dispase (1 U/ml; Sigma-Aldrich) was carefully injected. The trachea was then cut, and the lung was excised and rinsed with sterilized PBS. The lung was incubated with the injected 2 ml dispase at room temperature for 45 min. The lung tissue was transferred into a dish containing DMEM with 0.1 mg/ml DNase (Sigma-Aldrich). The lung tissue was mechanically fragmented with tweezers and incubated for 10 min at room temperature while gently rocking on a rocker. The cell suspension was filtered through nylon mesh filters with 70 and 30 μm pores. The filtrate was then transferred into 50 ml tubes, centrifuged at 160 × *g* for 15 min at 4 °C, and the supernatant was aspirated. The cell pellet was collected and suspended in 2 ml erythrocyte lysis buffer (150 mM NH_4_Cl, 10 mM KHCO_3_, pH 7.2, and 0.1 mM EDTA). The lysis step was quickly terminated by addition of 13 ml DMEM. The resulting solution was transferred to a 15 ml tube and centrifuged at 160 × *g* for 12 min at 4 °C. The cells were suspended in 3 ml primary antibody cocktail that was prepared in DMEM containing PE-coupled mouse CD11b and CD45 or APC-coupled mouse F4/80 and CD44c antibody (BioLegend, San Diego, CA, USA). For staining, the cells were incubated for 10 min in the dark at 4 °C. The cells were then washed with DMEM and centrifuged at 160 × *g* for 10 min at 4 °C. The cells were resuspended in 1 ml DMEM and pre-filtered through a 50 μm filter into a tube for cell sorting. Cell sorting was done by FACS (BD Bioscience, San Jose, CA, USA) using a 100 μm nozzle. Double-negative cells were collected and sorted into type I or type II AECs based on size. The cells were then centrifuged at 160 × *g* for 10 min at 4 °C. Cells were resuspended in either culture-medium or buffer as required for subsequent analysis.

### Histology and immunohistochemistry

Lung tissues were fixed with 4% paraformaldehyde, embedded in paraffin, and sectioned at a 6-μm thickness for subsequent staining. For histological analysis, sections were stained with hematoxylin and eosin and Masson’s Trichrome stain kit (Sigma-Aldrich). For immunohistochemistry analyses, sections were subjected to antigen retrieval by heating for 20 min at 100 °C in 0.01 M sodium citrate (pH 6.0) and exposure to 3% H_2_O_2_ before incubation with primary antibodies, including α-SMA (1A4; 1:400 dilution; Sigma-Aldrich), fibronectin (1:500 dilution; DAKO), or ESRP1 (27A12; 1:200 dilution; Abcam). Immune complexes were detected using the Vectastain Elite kit (Vector Laboratories, Burlingame, CA, USA) and 3,3′-diaminobenzidine; the sections were counterstained with hematoxylin.

### Proteomic analysis for TM4SF5- and CD44-binding proteins

SNU761 cells were transiently transfected with control or Strep-tag conjugated TM4SF5 plasmid for 48 h and extracted using a Brij58-containing lysis buffer. Lysates were immunoprecipitated using streptavidin-agarose bead (Thermo Fisher Scientific) prior to separation by SDS-PAGE. After in-gel trypsin digestion of proteins, peptides were analyzed using an LTQ XL linear trap mass spectrometer (Thermo Scientific) equipped with a nano-HPLC system (Eksigent, Dublin, CA, USA). NCI-H727 cells were transiently transfected with control or FLAG-tag conjugated CD44s or CD44v8-10 plasmids for 48 h, and then extracted using a Brij58-containing lysis buffer. Lysates were immunoprecipitated with FLAG M2 Affinity Gel (Sigma-Aldrich). After in-gel trypsin digestion, peptides were analyzed using LC-MS/MS. Tandem mass spectra were analyzed using the SEQUEST module of Proteome Discoverer (Thermo Fisher Scientific; version 1.4.1.14) and X! Tandem (The GPM, thegpm.org; version CYCLONE 2010.12.01.1). SEQUEST was set to search the protein database downloaded from the UniProt protein database using the keyword Homo sapiens (downloaded 15 April 2014, total of 124,318 entries). The digestion enzyme was assumed to be trypsin. X! Tandem was set to search a subset of the database also assuming trypsin as the digestion enzyme. SEQUEST and X! Tandem were searched with a fragment ion mass tolerance of 0.50 Da and a parent ion tolerance of 1.00 Da. Scaffold (Version Scaffold_4.4.1.1; Proteome Software Inc., Portland, OR, USA) was used to validate MS/MS based peptide and protein identifications.

### Statistical analysis

Data were shown as values (mean ± standard deviation) and analyzed by ANOVA with Tukey’s range-test or two-tailed unpaired Student’s *t*-test to determine the significance of difference between groups using GraphPad Prism version 7. The *p*-values less than 0.05 were considered statistically significant.

## Supplementary information

Supplementary Information
