# Supplementary material for: TM4SF5-mediated CD44v8-10 splicing variant promotes survival of type II alveolar epithelial cells during idiopathic pulmonary fibrosis
Source: Cell Death Dis. 2019 Sep 9;10(9):645. doi: 10.1038/s41419-019-1878-5 (PMC6733838; doi:10.1038/s41419-019-1878-5)
Supplement: Supplementary file 1 — Supplementary Information [file 41419_2019_1878_MOESM1_ESM.pdf]

TM4SF5-mediated CD44v8-10 splicing variant promotes survival of type II alveolar epithelial cells during idiopathic pulmonary fibrosis

Ji Eon Kim[1], Hye-Jin Kim[1], Jae Woo Jung[2], Dae-Geun Song[1,3], Dasomi Park[1], Haesong Lee[1], Hyejin Um[1], Jinsoo Park[1], Seo Hee Nam[1], Moonjae Cho[4], and Jung Weon Lee[1,2,5].

Supplementary Table S1 and Figures S1 to S5 are included

Table S1. The list of proteins that bind to both TM4SF5 and CD44 by two separate proteomic analysis.

Proteins bind to both TM4SF5 and CD44	MW (kDa)
Calnexin	73
Transferrin receptor protein 1	85
**4F2 cell-surface antigen heavy chain, CD98**	**58**
Integrin beta-1	88
Exportin-2	110
Trifunctional enzyme subunit alpha, mitochondrial	83
Integrin alpha-1	131
Sodium/potassium-transporting ATPase subunit beta-1	35
Integrin alpha-2	129
Claudin-1	23
Signal recognition particle receptor subunit beta	30

Figure S1

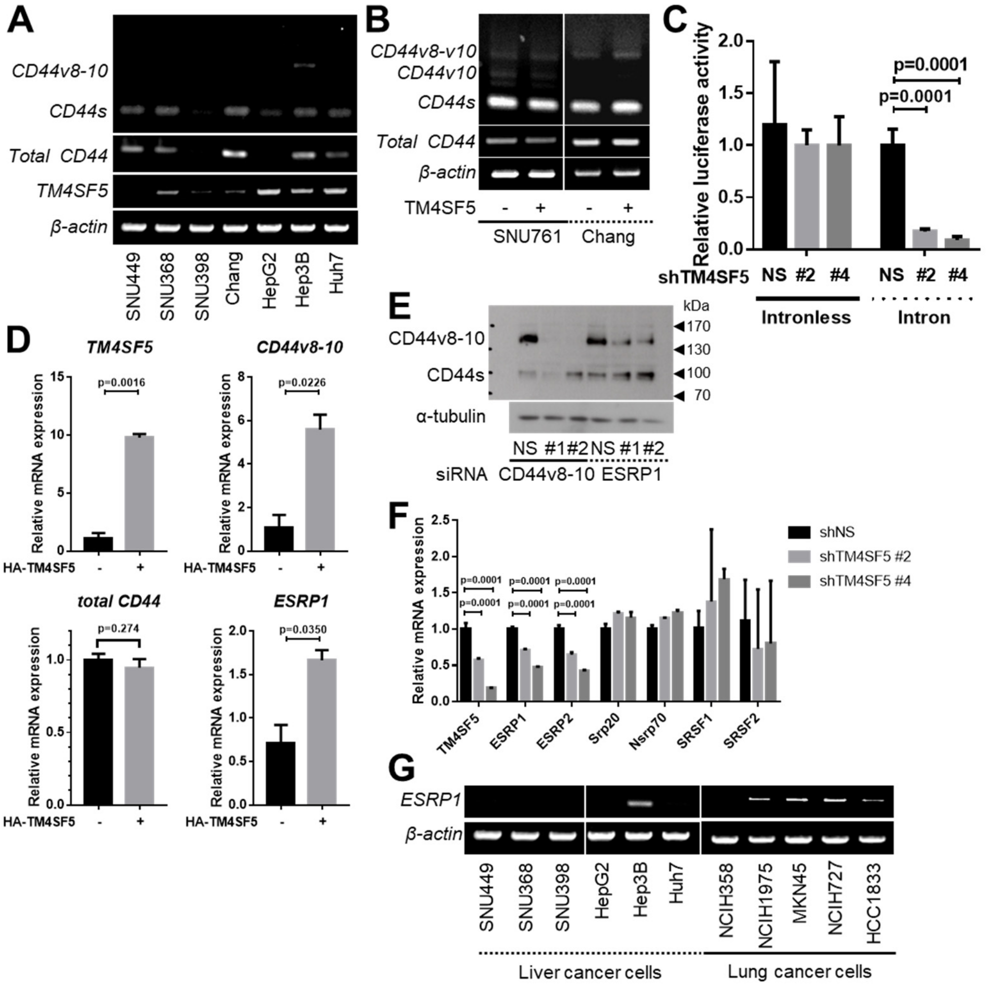

Figure S1. Molecular linkage from TM4SF5 to CD44v8-10 via Zeb2/ESRPs in lung, but not liver, epithelial cells. (A and B) mRNA levels of *CD44s* and splicing variants in liver epithelial cells were irrelevance to *Tm4sf5* mRNA levels. (C) Intron release was analyzed using a splicing luciferase reporter system with intronless or intron-containing pre-mRNA constructs in TM4SF5-positive and TM4SF5-suppressed NCI-H727 cells. (D) qRT-PCR of different mRNAs upon TM4SF5 expression in NCI-H358 cells was performed. (E) NCI-H727 cells without or with CD44v8-10 or ESRP1 suppression were processed to immunoblots. (F) NCI-H727 cells without or with TM4SF5 suppression (F) or liver and lung epithelial cells (G) were processed to qRT-PCR for the indicated mRNAs. *p* values were calculated by two-tailed unpaired Student's *t*-test. *p* values less than 0.05 were considered statistically significant. Data represent three independent experiments. See also Figure 1.

Figure S2

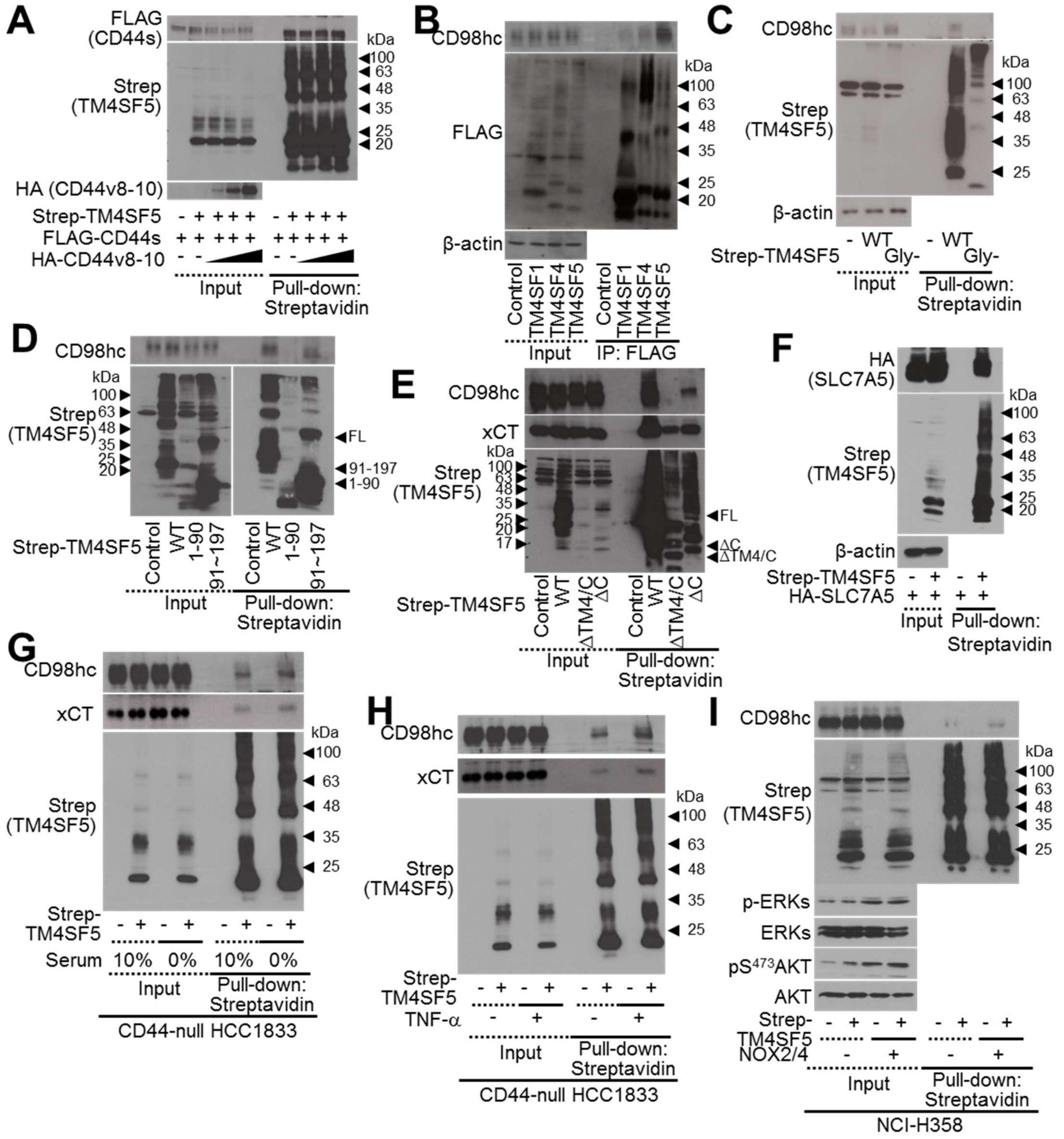

Figure S2. The characteristics of the protein complex of TM4SF5 with CD44 variant, CD98hc, and xCT. (A to F) CD44-positive NCI-H358 cells were transfected with indicated expression plasmids, before whole cell lysate harvests and pulling-down using streptavidin-agarose beads. The lysates (input) and co-precipitates were immunoblotted in parallel. (G to I) CD44-null HCC1833 cells (G and H) or CD44-positive NCI-H358 cells (I) were without or with serum-starvation (10% or 0%, respectively, G), TNF-α treatment (H), or NOX2/NOX4 (NOX2/4) transfection (J), before lysate (input) harvests and immunoblottings. Data represent three independent experiments. See also Figure 2.

Figure S3

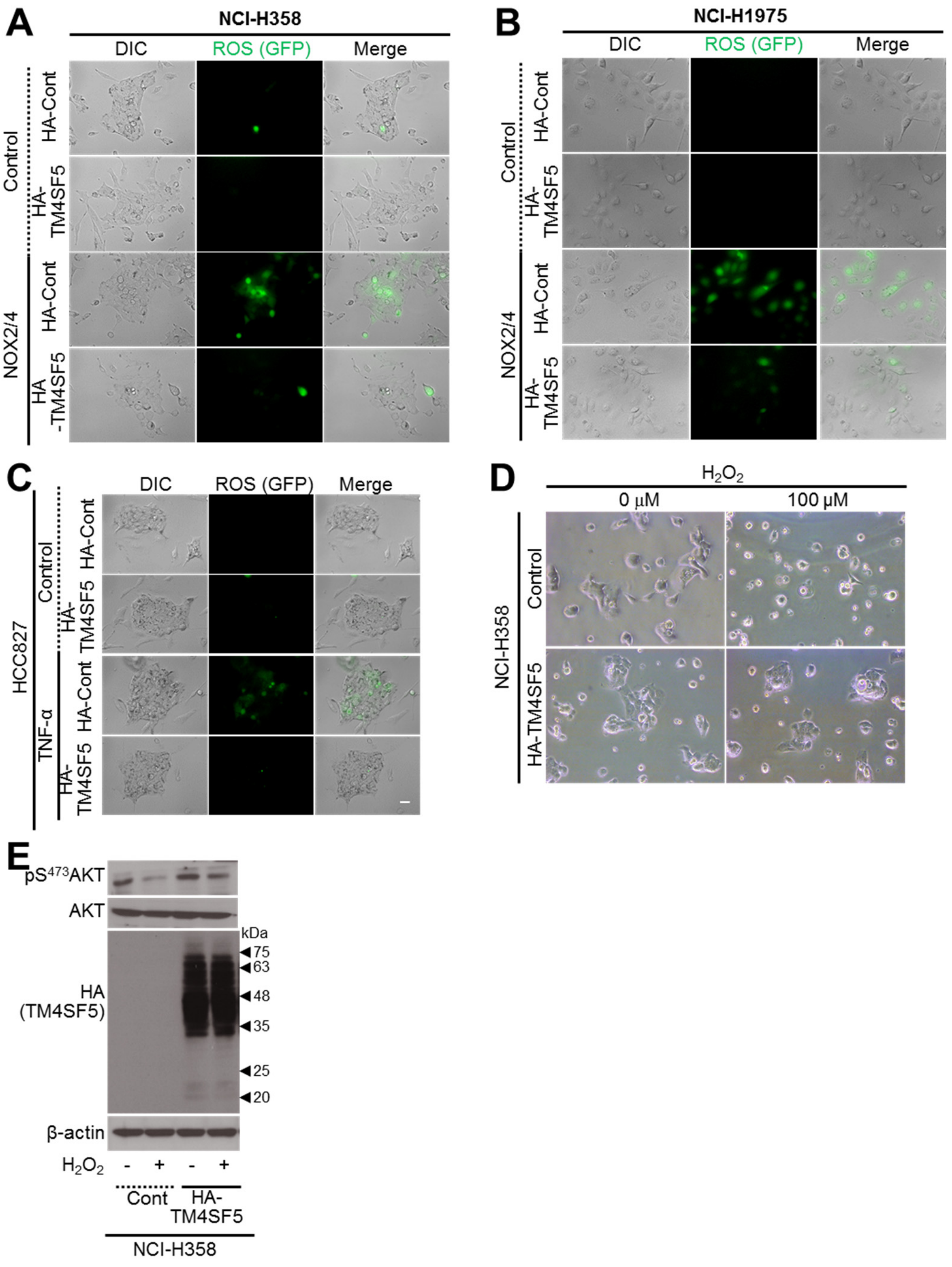

Figure S3. Lung epithelial cells showed survival under ROS-accumulating conditions, depending on TM4SF5 expression. Cells showed TM4SF5-dependent less accumulation of ROS even after NOX2/NOX4 (NOX2/4) expression (A and B) or TNF-α treatment (C). (D and E) NCI-H358 cell without (0 or -) or with H_2O_2 (100 μM or +) for 24 h, before phase contrast imaging to show cellular roundup (and presumably thereby death, D) and immunoblots for pS^{473}Akt to see survival signaling activity (E). Data represent three independent experiments. See also Figure 3.

Figure S4

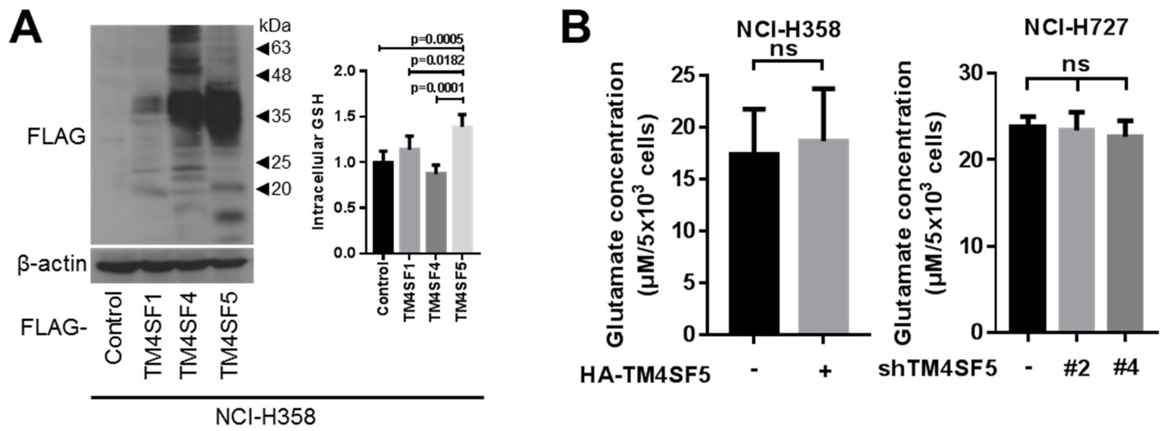

Figure S4. TM4SF5-mediated xc^{-} system. (A) NCI-H358 cells were transfected with expression plasmids for either TM4SF1, TM4SF4, or TM4SF5, before immunoblottings (left) or analysis of intracellular GSH levels (right). (B) Cells transfected with HA-TM4SF5 plasmid or shRNA targeting a control (shNS) or TM4SF5 sequence (#2 or #4, Table 1) were collected and harvested for whole cell lysates, before analysis of glutamate levels from the whole cell lysates. The *p* values were analyzed by ANOVA with Tukey's post-test. *p* values less than 0.05 were considered statistically significant. NS depicts non-significance. Data represent three independent experiments. See also Figure 4.

Figure S5

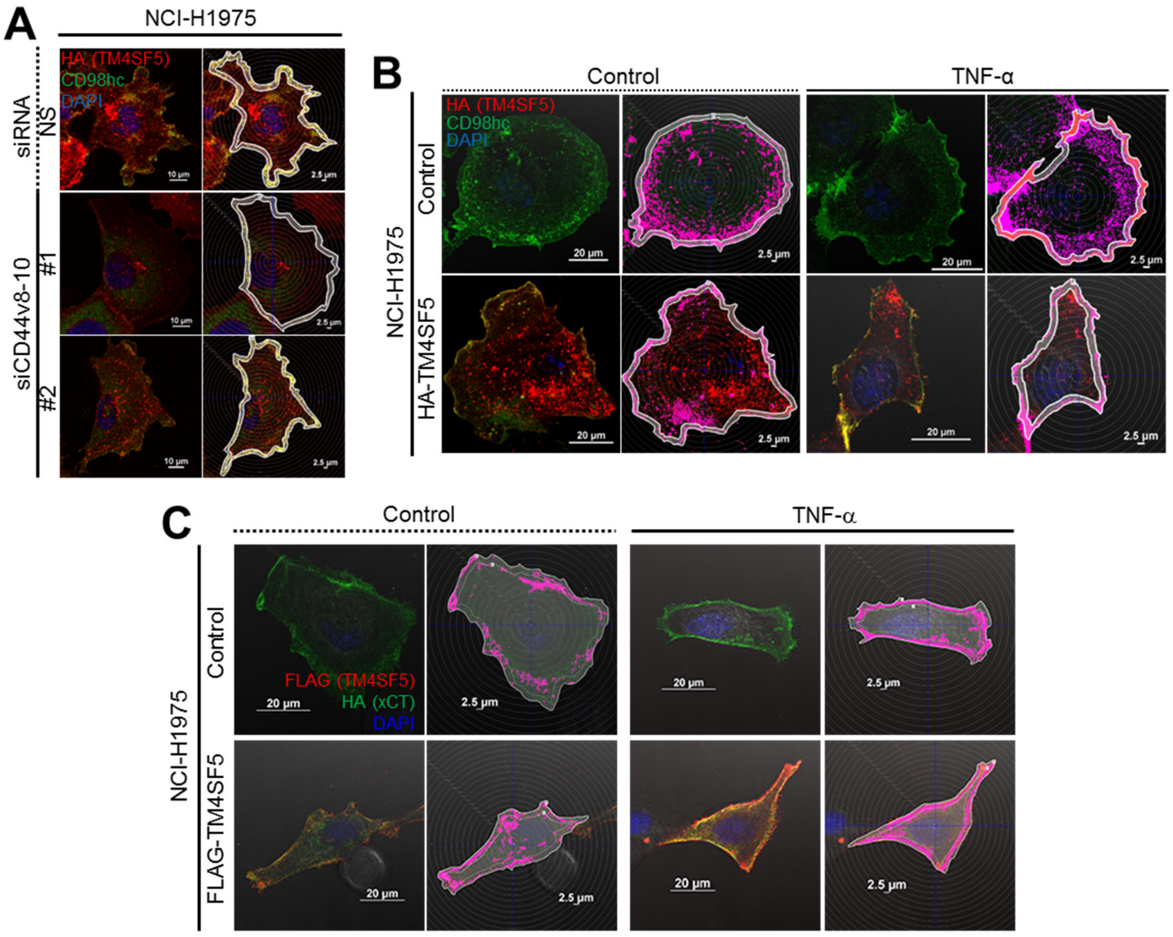

Figure S5. Approaches to determine translocalization of CD98hc or xCT to plasma membrane (-proximal) regions upon TM4SF5 expression. NCI-H1975 cells were transfected with shRNA for a control sequence (shNS) or sequences for CD44v8-10 (#1 and #2, Table 1) (A), with control or HA-TM4SF5 expression plasmids (B), or with control or FLAG-TM4SF5 expression plasmids (C) for 48 h, before immunofluorescent imaging to determine translocalization of each molecule. Images were saved using a C2+ confocal microscope (Nikon) with a normal PMT (Nikon) and a CFI Apochromat Lambda S 60× NA1.49 oil immersion objective (Nikon) after excitation with 405 nm, 488 nm, and 561 nm laser lines. NIS software (Nikon) was used to calculate areas with molecules. The active total regions of interest (ROIs) were defined to reflect the DIC image of individual cells at 2.5-μm intervals, and ROI regions for the red and green channels were converted to binary values that were exported to Excel. See also Figure 4.
